# Resilience in mental health: linking psychological and neurobiological perspectives

**DOI:** 10.1111/acps.12095

**Published:** 2013-03-14

**Authors:** B P F Rutten, C Hammels, N Geschwind, C Menne-Lothmann, E Pishva, K Schruers, D van den Hove, G Kenis, J van Os, M Wichers

**Affiliations:** 1 Department of Psychiatry and Psychology, Faculty of Health, Medicine and Life Sciences, School for Mental Health and Neuroscience (MHeNS), European Graduate School of Neuroscience (EURON), Maastricht University Medical CentreMaastricht, the Netherlands; 2 Research Group on Health Psychology, CLEP, Department of Psychology, University of LeuvenLeuven, Belgium; 3 Center for Learning and Experimental Psychology, Catholic University of LeuvenLeuven, Belgium; 4 Department of Psychiatry, Psychosomatics and Psychotherapy, University of WürzburgWürzburg, Germany; 5 King's Health Partners, Department of Psychosis Studies, Institute of Psychiatry, King's College LondonLondon, UK

**Keywords:** resilience, mental health, psychiatry, epidemiology, neurobiology

## Abstract

**Objective:**

To review the literature on psychological and biological findings on resilience (i.e. the successful adaptation and swift recovery after experiencing life adversities) at the level of the individual, and to integrate findings from animal and human studies.

**Method:**

Electronic and manual literature search of MEDLINE, EMBASE and PSYCHINFO, using a range of search terms around biological and psychological factors influencing resilience as observed in human and experimental animal studies, complemented by review articles and cross-references.

**Results:**

The term resilience is used in the literature for different phenomena ranging from prevention of mental health disturbance to successful adaptation and swift recovery after experiencing life adversities, and may also include post-traumatic psychological growth. Secure attachment, experiencing positive emotions and having a purpose in life are three important psychological building blocks of resilience. Overlap between psychological and biological findings on resilience in the literature is most apparent for the topic of stress sensitivity, although recent results suggest a crucial role for reward experience in resilience.

**Conclusion:**

Improving the understanding of the links between genetic endowment, environmental impact and gene–environment interactions with developmental psychology and biology is crucial for elucidating the neurobiological and psychological underpinnings of resilience.

**Summations**

- Resilience is not merely characterized by the absence of psychopathology but is the dynamic process that enables the individual to successfully adapt to severe adversity over the life course.
- Resilience entails both the process of preventing or attenuating health disturbance after adversity, and the process of swift recovery from adversity-related mental ill health.
- Understanding the psychology and neurobiology underlying resilience will help develop strategies aimed at preventing psychopathology after exposure to severe adversity.

**Considerations**

- Secure attachment, experiencing positive emotions and having a purpose in life are three important psychological building blocks of resilience.
- Improving the understanding of the links between genetic endowment, environmental impact and gene–environment interactions with developmental psychology and biology is crucial for elucidating the neurobiological and psychological underpinnings of resilience.
- While animal research has mostly focussed on sustainability, i.e. the prevention of mental health disturbance, human resilience studies have also investigated determinants of recovery from adversity-related mental ill health.
- To improve the understanding of the neurobiological underpinnings of resilience, translational human and animal studies should attempt to define behavioural outcome measures that can be investigated across species.

## Introduction

Research on mental health has historically been dominated by investigations of risk and vulnerability for developing mental ill health. An important paradigm shift is currently underway as scientific work is moving its focus from the factors and mechanisms that determine vulnerability to mental ill health, to the factors and mechanisms that stimulate individuals to remain healthy or to recover swiftly when facing severe adversities over the course of life. In this framework, resilience is considered the outcome of the successful adaptation to severe adversity over the life course. For example, although traumatic experiences during childhood are a well-known risk factor for various psychiatric disorders with convincing evidence from epidemiological studies suggesting a causal relation between childhood trauma and various psychiatric disorders in adulthood 1, many children exposed to severe trauma do not develop psychopathology but can adapt successfully (i.e. sustainability) or recover swiftly 2. The factors and mechanisms that modulate and mediate an individual's risk and resilience can be studied at different levels ranging from more general levels such as the society that a person is living in, or the more direct social surrounding of an individual (peer group, neighbourhood), to more individual levels such as the individual's psychological abilities and the molecular and cellular biology of an individual's neuronal circuitry. The present manuscript selectively reviews the psychological and biological studies at the individual level of resilience; the reader is referred to other review articles and a recent book on this topic, see e.g. 3–5, for information on resilience defined at other levels. The present manuscript furthermore does not review the entire literature on interventions to promote resilience at the individual, nor discusses interventions at the population level.

### The concept of resilience; trajectories of risk and resilience

**Fig. 1:**
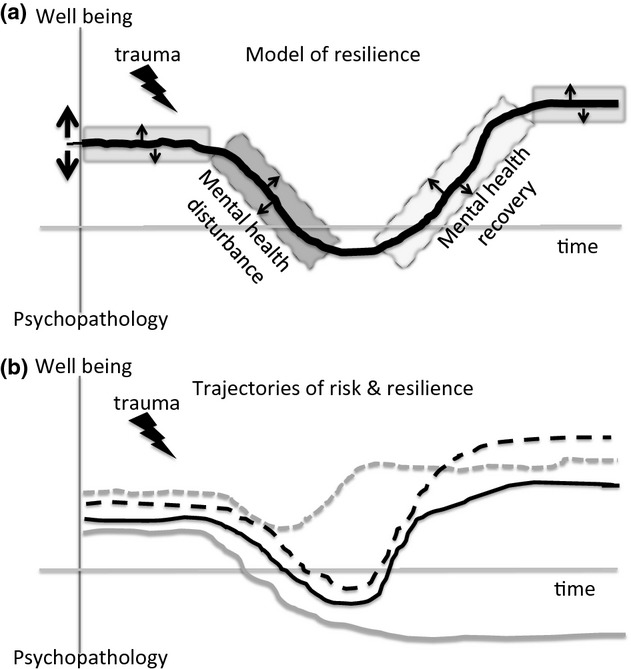
Model of resilience (a) and trajectories of risk and resilience (b). (a) Provides a model for illustrating the level of an individual's mental wellbeing over time and illustrates a decline of mental wellbeing in response to a severe adversity such as exposure to trauma followed by recovery in mental wellbeing. An individual may vary in i) the level of mental wellbeing before the exposure, ii) The speed and severity of mental health disturbance in response to the exposure, iii) The speed and timing of mental health recovery and iv) level of mental health and wellbeing after the exposure-related disturbance and recovery. (b) four different trajectories (grey full, grey dashed, black full and black dashed lines) of individuals' risk and resilience for developing psychopathology in response to exposure to a severe stressor/trauma. The grey full line depicts an individual with a positive level of mental health preceding the exposure, a consistent decline in mental health following the exposure without subsequent recovery. The grey dashed line depicts an individual with a positive level of mental health preceding the exposure (a more positive mental health than the others), with a temporary and relatively brief decline in mental health following the exposure followed by swift recovery up to a somewhat higher level of mental health than before the exposure. The full black line depicts an individual with a positive level of mental health preceding the exposure, a consistent decline in mental health following the exposure that recovers quickly to preexposure levels of mental health after a certain delay period in which the individual expresses psychopathology. The dashed black line depicts an individual with a positive level of mental health preceding the exposure, a consistent decline in mental health following the exposure that recovers quickly to preexposure levels of mental health and continues to increase thereby surpassing preexposure levels of mental health (this can be seen as post-traumatic growth).

Human and animal studies on ‘resilience’ have used different definitions and measurements of resilience, and the variable measurements and definitions of resilience preclude a meta-analysis. Successful adaptation and swift recovery after experiencing severe adversity during life currently represents a generally accepted definition of resilience 6. Resilience is thus as a dynamic and adaptive process that subserves maintaining, or swiftly regaining, homeostasis in conditions of stress. As illustrated in Fig. 1a, this concept of resilience may encompass several processes. As discussed previously, e.g. by Davydov et al. 6, the concept of resilience entails on one hand a process of sustainability that prevents and attenuates disturbance of mental health and wellbeing after exposure to severe adversity, and on the other hand a process of rapid recovery from mental health disturbance following exposure to adversity. Figure 1a provides a model for illustrating the level of an individual's mental wellbeing over time and illustrates that an individual may vary in i) the level of mental wellbeing before the exposure, ii) the speed and severity of mental health disturbance in response to the exposure, iii) the speed and timing of mental health recovery and iv) level of mental health and wellbeing after the exposure-related disturbance and recovery. Thus, as illustrated in Fig. 1b, one can envision many different trajectories of individuals' risk and resilience for developing psychopathology in response to exposure to a severe stressor/trauma, ranging from a trajectory showing consistent decline in mental health following exposure to adversity without subsequent recovery of mental health for a prolonged period of time, to a decline in mental health following the exposure that recovers quickly to preexposure levels of mental health and continues to increase thereby surpassing preexposure levels of mental health. This latter response, known as post-traumatic growth, is a very interesting form of adaptation, in which the individual may have obtained a better understanding of his life, possibly from a new perspective, or may have learned to respond efficaciously to similar challenges in the future 5.

The determinants and mechanisms of the different aspects of this concept of resilience may well differ from each other, i.e. those determinants and mechanisms underlying the set point of mental health can be different from those underlying mental health disturbance or those underlying mental health recovery or post-traumatic growth. Thus, a range of complexly interacting factors determines the final outcome of a ‘resilient’ phenotype. However, as discussed below, some of the factors that have been associated with distinct aspects of resilience may have a positive influence on various (if not all) processes involved in resilience.

### Aims of the study

We aim to review the current state of the literature on the psychological and neurobiological factors and mechanisms that may underlie resilience in a selective fashion (i.e. without attempting to provide a full and complete overview). To facilitate the understanding of the psychological and neurobiological findings on resilience, we start by briefly reviewing the neurobiological circuitry involved in the stress response and in reward processes, and with introducing the concepts of gene–environment interaction and experience-dependent plasticity. After describing the findings from animal studies on resilience to stress, and summarizing the findings of human resilience, with a particular emphasis on secure attachment, positive emotions, and having a purpose in life, we attempt to integrate findings from human and animal studies on resilience, discuss the current challenges in the field of resilience research and suggest avenues for future work.

## Material and methods

Electronic and manual literature searches of MEDLINE, EMBASE and PSYCHINFO were performed, complemented by review articles and cross-references. The search was limited to articles published before August 2012 (without early date constraints). The search consisted of terms around neurobiological background of resilience, animal studies on resilience and human studies on resilience. Titles and abstracts, when available, were reviewed to exclude irrelevant studies, and the identified manuscripts were evaluated by at least two authors independently. In selecting human studies on psychological aspects of resilience, we primarily focussed on articles covering the topics of secure attachment, positive emotions and purpose of life.

## Results

### Neurobiological background

#### Neurocircuitries

**Fig. 2:**
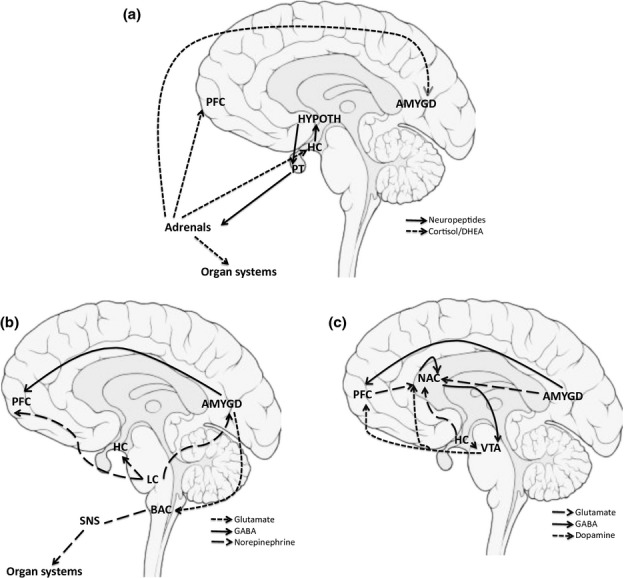
Brain circuitries involved in the stress response and reward experience. Key brain regions involved in the response to stress and reward experience. (a) Hypothalamus–pituitary–adrenal axis. Psychological and physiological stressors are known to activate the hypothalamus–pituitary–adrenal (HPA) axis, leading to corticotrophin releasing hormone (CRH) production by the hypothalamus and adrenocorticotropic hormone (ACTH) release from the anterior pituitary (indicated by the black arrows). ACTH induces glucocorticoid hormone release from the adrenal cortex into the circulation (indicated by the grey arrows). Moreover, GCs exert a negative feedback on the activation of the HPA activation, via GR in the hippocampus, therefore controlling their own release. Cortisol has important regulatory functions on the amygdala (AMYGD), hippocampus and prefrontal cortex (indicated by the grey arrows). Besides cortisol, another adrenal steroid hormone released under stress is (dehydroepiandrosterone) DHEA (indicated by dashed arrows). DHEA is released synchronously with cortisol from the adrenal glands. It has antiglucocorticoid and antiglutamatergic characteristics in the brain, and is – in general – related to inhibition of the HPA axis. (b) Norepinephrine and sympathetic nervous system (SNS).Next to activation of the HPA axis, stress increases norepinephrine release from the LC (locus coeruleus) to its projecting neurons in the amygdala, PFC and hippocampus (indicated by the long dashed arrows). As a result, the PFC is inhibited both by the LC itself and the amygdala (indicated by the black arrow), thereby favouring instinctive responses over complex thinking. Moreover, the amygdala stimulates brainstem autonomic centres (BAC). During stress, the sympathetic autonomous nervous system (SNS) releases epinephrine and NE. (c) Mesolimbic reward pathway. Activation of the hippocampus, amygdala and PFC also activates the mesolimbic reward pathway. These three structures have glutamatergic projections to the (nucleus accumbens) NAC (indicated by the long dashed arrows). The strength of the synapse is modulated by dopamine signalling that modulates glutamate release. A reward stimulus leads to phasic dopamine release from the VTA (indicated by short dashed arrows). GABAergic neurons in the NAc in turn exert negative feedback on the VTA, thereby controlling dopamine release, and dopaminergic signalling to the PFC. Integration of signals of the VTA, hippocampus (learned behaviour) and amygdala (emotional behaviour) by the PFC underlies the sensation of the reward feeling. In addition, BDNF is produced in the VTA and transported to the NAc via its dopaminergic afferent. It is likely that BDNF, when administered in the VTA-NAc also activates the GABAergic neurons in the NAc, thus inhibiting dopaminergic input from the VTA, possibly underlying blunted responses to emotional stimuli or symptoms of anhedonia.

The neurocircuitries mediating the stress response and reward experience are thought to be crucially involved in the neurobiology of resilience. The efficiency in activating and terminating the response to stress is regulated by elaborate negative feedback systems in the brain and the rest of the body. An appropriate response to stress is a prerequisite for sustained health in the face of adversities, and thus for reducing mental health disturbance after exposure to severe adversities. The hypothalamus–pituitary–adrenal (HPA) axis, the sympathetic nervous system (SNS) and the dopaminergic and serotonergic neurotransmitter systems are major neural systems that govern the stress response, and these systems are illustrated in Fig. 2. Activation of the HPA axis leads to corticotrophin releasing hormone (CRH) production by the hypothalamus and adrenocorticotropic hormone (ACTH) release from the anterior pituitary. ACTH induces glucocorticoid hormones (GC; cortisol in humans, corticosterone in rodents) release from the adrenal cortex into the circulation, and these circulating GCs have effects on multiple organ systems via signalling cascades mediated by glucocorticoid receptors (GR) and mineralocorticoid receptors that are expressed in virtually all tissues of the body. Although short-term cortisol elevations promote adaptive behaviour and are therefore protective, long-term hypercortisolemia is considered harmful, as it, for example, impairs neurogenesis. The neurocircuitry mediating reward experiences revolve around activation and regulation of mesolimbic dopaminergic projections from the Ventral Tegmental Area (VTA) to the nucleus accumbens (NAc) (see Fig. 2). Mesolimbic neurotransmission encompasses the NAc as the site of integration of bottom-up sensory experiences with top-down cognitive control of dopamine neurotransmission, thus regulating dopamine firing in the VTA via GABAergic feedback through the ventral pallidum. The activity of the mesocorticolimbic circuit is furthermore subserved by reciprocal connections with limbic and frontal cortical areas.

#### Gene–environment interactions

According to the concept of genetic moderation of sensitivity to the environment, differences in genetic endowment explain why people respond differently to the same environment. Most evidence for gene–environment interactions (GxE) has come indirectly from twin and adoption studies, and a variety of naturalistic designs in which non-specific genetic contributions have been assessed. More recently, researchers have obtained information about how variation in specific measured genes interacts with specific measured environments 7. Genetic moderation of environmental sensitivity gives rise to *synergism*, or *interaction*, as the biological effects of G and E are dependent on each other in such a way that exposure to neither or either one alone does not result in the outcome in question, whereas exposure to both does. For example, stressful life events have been documented to increase risk for psychiatric illness in carriers of distinct variants of genes such as 5HTT, FKBP5 and CRH1 but not in carriers of the alternative variants of these same genes [see below, and reviewed recently by Feder et al. 8].

#### Experience-dependent plasticity

The brain enables an individual to respond with the appropriate behaviour to various stimuli. This requires dynamic adaptations in the molecular and cellular processes that represent the pathway from sensory perception to behavioural responses. Recent work has suggested the involvement of distinct biological mechanisms that mediate and moderate the imprinting of experiences. These experience-dependent mechanisms regulate the sensitivity and plasticity of the central nervous system and act at several biological levels (likely partly in parallel with each other): i) cellular changes such as neurogenesis, pruning and sprouting of synapses, myelination of axons and alterations to the number of dendritic spines^9^, ^10^, ii) subcellular changes, such as alterations to the cytoskeleton and the extracellular matrix and changes in the levels of intracellular signalling molecules 11 and iii) molecular (epi) genetic changes such as DNA methylation and chromatin changes 12. Thus, one can envision that aberrant regulation at any of these levels may moderate risk for and resilience to the consequences of stress and that resilience thus depends on a range of environmental and genetic factors during life.

Research has shown that epigenetic regulation of gene transcription is a key mechanism subserving adaptation to external stimuli at the molecular level 13. Epigenetics refers to the reversible regulation of various genomic functions, occurring independently of DNA sequence and principally mediated through changes in DNA methylation and chromatin structure. Epigenetic mechanisms include modifications in response to environmental stimuli, and contribute to cell- and tissue-specific gene expression profiles during brain development involving processes such as neurogenesis and synaptic plasticity 14, 15. Recent animal studies have demonstrated that the development of the brain and the functional abilities of the brain such as memory formation, learning, motivation and reward are all regulated by epigenetic regulation of gene expression 16, 17. Epigenetic mechanisms are furthermore proposed to play crucial roles in ageing and age-related neurodegeneration 18, 19. Thus, epigenetic mechanisms appear to be fundamentally involved in the neurobiological processes underlying individual variation in response to experiences and environmental exposures during development and ageing 20.

### Animal research on resilience

To date, almost all experimental animal studies on resilience to stress have focused on measuring (differential) disturbances in normal behavioural patterns after exposure to stress. Although human resilience phenotypes in animals (positive aspects of wellbeing) are very difficult to identify and assess, it is possible to measure successful adaptation after exposure to severe adversity in animals or the rate of recovery from adversity-related behavioural changes. Unfortunately, the rate of recovery after exposure to stress has hardly been investigated at all in animal studies and therefore cannot be discussed in detail here. The effects of stress have been investigated using a range of experimental paradigms in various phases during life of various animal species. Most animal research thus far has used experimental paradigms that may model disruption in secure social attachment using exposures such as prenatal maternal restraint stress, maternal deprivation in early life, maternal nurturing behaviour, social isolation and chronic social defeat stress.

#### Early rearing conditions

Recent experimental animal research has shown that the psychosocial environment, and stress in particular, can mediate changes in gene expression and behaviour during key developmental periods. A first important line of research, conducted by Michael Meaney and colleagues, has shown that parental care during early life induces long-term changes in behaviour as well as in gene expression mediated by epigenetic changes in the hippocampus of rats 21. As compared with offspring of mother rats with low-nurturing behaviour, offspring of high-nurturing mother rats (displaying more licking and grooming behaviour) were less anxious, had attenuated corticosterone responses after stress exposure and expressed higher levels of the glucocorticoid receptor (GR) in the hippocampus in adulthood 22. Interestingly, the methylation level of the promoter region of *Nr3c1*, i.e. the gene encoding the GR, was elevated already the first week of life in the hippocampus of pups that received less and lower quality nurturing 23, an effect that persisted into adulthood. Subsequent experiments demonstrated that manipulation of epigenetic mechanisms could reverse these gene expression alterations, as well as the related behavioural phenotypes in adult offspring 23. Detailed molecular analyses of the hippocampus of these animals suggested that the parenting style during early life impacted on many genes (and not only on *Nr3c1*) throughout the life course. These findings clearly show that environmentally induced epigenetic changes occur during brain development and may generate individual differences in stress vulnerability. In addition to the study of Meaney, other studies have shown that the mother–infant interaction has long-lasting effects on endocrine and behavioural responses later in life 24.

Another interesting line of research has explored the effects of maternal separation on biology and behaviour. Although most studies observed detrimental effects of maternal separation, studies where rat pups were separated from their mother for a very brief period, i.e. 15 min, indicated that these pups, compared with non-separated pups, were more stress resistant later in life. Interestingly, as compared with offspring not separated from their mother for these brief spells in very early life, animals with brief spells of maternal separation showed higher levels of glucocorticoids (GCs) directly after stress exposure in adulthood, with a fast return to basal levels 25. Thus, type, severity and/or duration of stressful experience early in life seem to influence differential stress reactivity later in life.

The effects of manipulations of mother–offspring interactions depend, at least in part, on permanent changes in the brain regions that have a pivotal role in regulation of the stress response, such as the hippocampus and the hypothalamus 26. A number of studies have suggested that neurotrophins (i.e. signalling molecules that in general promote neurogenesis, strengthen synaptic contacts, enhance cell survival and are therefore thought to enhance plasticity) are attractive candidates for mediating these persistent changes. Indeed, maternal separation increases nerve growth factor (NGF) expression in the hippocampus, cerebral cortex and hypothalamus 27. Manipulations of mother–offspring interactions in rodents are furthermore known to induce acute elevations of brain-derived neurotrophic factor (BDNF) in the hippocampus and prefrontal cortex (PFC), with the same animals showing reduced BDNF expression in the same brain regions during adulthood 24.

A series of experiments has demonstrated similar effects of early experience in macaque monkeys, i.e. primates. Comparing behavioural and biological read outs from peer-reared macaque monkeys with those from mother-reared macaque monkeys [reviewed in 28] demonstrated that parenting behaviour during early life is crucial for the development of an appropriate stress response, reward experience and social interactions in adulthood and that these effects depended on genetic background. For example, the 5-HTTLPR genotype influenced the hormonal responses of the macaque monkeys during stress, while this influence was moderated by early experience 29.

#### Stress during adolescence

One of the most successful experimental paradigms testing for differential disturbance in normal behaviour after exposure to chronic and severe stress during early adolescence is the social defeat paradigm 30. In the social defeat paradigm, male test mice aged 6–10 weeks (corresponding to puberty and adolescence in humans) are placed into the territory of a larger and more aggressive resident mouse. The mice are left in this physically and socially stressful situation for approximately 10 min, which leads to subordinate behaviour of the test mouse. After these 10 min, the mice remain in sensory (but not physical contact) with each other for the rest of the day, and the procedure is repeated for 10 consecutive days. The experimental paradigm is known to induce anxiety-like behaviour, prolonged elevations in corticosterone levels and a range of other molecular and cellular changes 31. Mice that were subjected to chronic social defeat stress furthermore showed a prolonged reduction in orexin signalling in the hypothalamus 32. Orexin has been implicated in arousal and feeding behaviour, but more recently also in the mesolimbic reward pathway. Orexin receptors are highly expressed in the VTA and activation of these receptors increases the firing rate of GABAergic and dopaminergic neurons in the VTA (see Fig. 2) 33. Although all mice have the same genetic background, and are exposed to similar conditions of social defeat, this experimental paradigm has repeatedly been shown to elicit two distinct responses in the domain of social behaviour: one group of mice displaying social avoidance after the social defeat experience (these mice are called ‘susceptible’), whereas a second group of mice still showing social interaction rates that are comparable with the control group (and is therefore called ‘unsusceptible’ or ‘resilient’) 34. Thus, only a distinct subpopulation (i.e. the ‘susceptible’ mice) displays social avoidance and behavioural signs of anhedonia, while all exposed animals (‘susceptible’ and ‘unsusceptible’ mice) show elevated corticosterone levels and increased anxiety-like behaviour 31.

The social defeat stress model has been proposed as a model for depression-like behaviour or for behavioural disturbances associated with post-traumatic stress disorder, while it is furthermore particularly useful for studying the neural basis and the molecular and cellular mechanisms underlying differential vulnerability or sustainability to the effects of chronic social defeat stress. These pioneering studies that investigate differential response to social defeat stress are thus very relevant to the topic of resilience. Although only a limited number of studies (i.e. two) have studied the differential response to social defeat stress using this paradigm, the findings of these studies have had a big impact in the field. For example, Krishnan et al. 35 observed that only susceptible mice showed increased BDNF protein levels in the NAc(which correlated with measures of an hedonic behaviour), whereas susceptible and unsusceptible mice did not differ in their corticosterone response to a severe stressor. As knock-down (inhibiting expression of a gene) of the *BDNF* gene in the VTA increased the proportion of ‘unsusceptible’ mice to the chronic social defeat paradigm, one could argue that BDNF signalling in dopaminergic neurons of the VTA may mediate the response to social defeat. Further detailed molecular analyses using microarray platforms revealed that voltage-gated K+ channels were specifically up-regulated in the VTA of ‘susceptible’ mice, and that this upregulation was connected to increased firing rates of dopaminergic VTA neurons 31. Thus, these findings illustrate that mesolimbic dopaminergic neurotransmission is centrally involved in the differential response to social defeat stress in mice.

#### Manipulations to attenuate disturbance after stress exposure in animals

Experimental animal studies are very instrumental in testing the effects of interventions on sensitivity to stress, and here we will briefly review findings on the effects of physical activity, enriched environment and a dietary intervention. Rodents with higher levels of physical activity have been shown to display attenuated effects of psychological stressors on depression-like behaviour and cognition 36, enhanced neuroplasticity 37 and an improved stress response 38. For example, voluntary wheel running has been shown to increase BDNF and NGF mRNA and protein levels in the hippocampus of rats, with the elevated BDNF levels persisting for several days after cessation of exercise 39. Wheel running before the stress paradigm (i.e. the forced swimming test) prevented stress-induced down-regulation of BDNF in the hippocampus of rats and attenuated depressive-like behaviour 40.

Although human studies clearly show that an extended social network and positive experiences are important factors contributing to resilience (see further), these aspects are difficult to model and to measure in animals. Nevertheless, animal research using environmental enrichment strategies, i.e. using social housing with plenty of possibilities for play, has suggested an important role for social contact and positive experiences in resilience to social defeat. For example, mice housed in an enriched environment have been shown to display an improved extinction of the submissive and depressive-like phenotype after social defeat stress exposure, improved cognitive performance in the Morris water maze test (a learning and memory task in which a rat needs to learn to orientate itself and navigate to a hidden platform based on spatial information) and increased NGF and BDNF levels in the hippocampus 41. Data showing that transgenic mice lacking normal neurogenesis do not display this antidepressant effect of environmental enrichment strongly suggest that adult neurogenesis mediates the beneficial effect of enriched environment 42.

Several studies have furthermore suggested that dietary interventions may ameliorate the effects of stress in animals. One dietary intervention known to have substantial impact on physical health and behaviour is a diet restricted in calories (leading to substantial reductions in particularly carbohydrate intake). Such a diet is known to extend the lifespan in a range of animal species including primates 43, prevent age-related epigenetic 44–46 and cellular changes 47 as well as age-related cognitive decline, while also protecting against stress and depressive-like symptoms in rodents 32. Although the exact mechanism exerting the plethora of effects remains largely unknown, it has been suggested by Lutter and colleagues that the protective effects of caloric restriction against stress and depressive-like behaviour are mediated via ghrelin and orexin in the brain 32, while caloric restriction may also act via increasing BDNF levels in the hippocampus and amygdala 48.

In all, experimental animal studies on resilience to stress have mostly focused on measuring disturbances in behaviour after exposure to stress by comparing a group of animals exposed to stress and a (control) group of animals not exposed to stress. Recent research efforts have, however, started to explore the mechanisms underlying differential responses to stress by investigating differential responses in the group of animals exposed to stress, and comparing susceptible to unsusceptible, i.e. resilient, animals as well as comparing both these groups to control animals that were not exposed to stress. The speed of recovery or positive adaptation in animals after stress exposure has thus far been underinvestigated. The animal research to date has highlighted early experiences, gene–environment interactions, the HHPA axis (re)activity, neurotrophins and the serotonergic and dopaminergic neurotransmitter systems as important factors that may mediate the differential sensitivity to stress, and therefore, by implication, to mental health disturbance.

### Human research on resilience

The current literature shows that the building blocks of resilience are not merely the positive ends of a continuum with risk, but that they are separate (biological and psychological) qualities of wellbeing and mental health that enable successful adaptation or swift recovery from life adversity. Here, we focus the review and discussion on three core psychological domains that have been associated with resilience: secure attachment, positive emotions and purpose of life. These domains may impact on resilience by enhancing mental health in general, by preventing or attenuating mental health disturbance after exposure to adversity, or by bolstering the recovery from adversity-related mental health disturbance.

#### Secure attachment

The first important source of resilience in a human's life is the attachment he develops with his primary caregiver in the very early years. Attachment behaviour has been described as proximity-, comfort- and security seeking when confronted with some kind of stress (Bowlby in 49). Most of all, *secure* attachment has been associated with resilience 50. In a secure attachment relationship, the infant learns to integrate cognitive and affective experiences into one mental representation. Based on the experiences of this relationship, it will learn to trust others and be confident that it will be protected in the case of threat 49. The base for secure attachment is created from the first moments in life by available and attuned parenting 51. However, it is not before the age of 6 months that the child forms a genuine attachment (Bowlby in 51). Secure attachment to parents or other primary caregivers remains important throughout childhood and adolescence 51.

The attachment relationship has been found to be influenced greatly by the caregiver's behaviour 50. A secure attachment has been shown to result from supportive, sensitive and responsive parenting that is attuned to the child's needs and behaviours 52, 53. Behavioural genetic studies with twins indicate that attachment is indeed the result of environmental influences only 54, 55. Sensitive and warm parenting in particular appears to be the largest determinant of attachment security 56 as it is the parents who provide the child with a safe and stimulating learning environment 53 from which it can explore the world. Further supporting the importance of parenting in attachment and resilience, several studies have indicated that early prevention strategies that promote sensitive and stimulating caregiving, stimulate the development of positive cognitive as well as behavioural outcomes in the child 57. Several meta-analyses in this area indicate that supporting and training parents to adopt a sensitive parenting style, delivered at moderate frequency is not only able to increase sensitive parenting but can also increase secure attachment in children 58, 59.

The psychological link between a secure attachment and resilience, however, is thought to be reflected by the internal working model the child derives from his or her attachment experiences. Internal working models start to emerge at the end of the first year of life and continue to evolve until the age of five, when internal working models about how social relationships work are more clearly manifested 51. Experiences and memories of the child are interpreted and processed in a way that they match with the internal working model 51. It is therefore that attachment security has a long-lasting impact on resilience and wellbeing 50. Based on parents' behaviour, the infant develops internal mental representations of interpersonal relationships 52. Securely attached children form internal working models in which the self is perceived as worthy, others are perceived as being available and reliable, and the environment can be experienced as challenging but manageable with support from others 50. Such a working model leads to effective self-regulation, the capacity to infer own and other's mental states, to manage social relationships 50 and to deal effectively with various stressors. In support of the importance of healthy internal working models in resilience, studies have shown that helping foster parents and other (second) caregivers to provide sensitive and supportive parenting resulted in similar effects as above interventions with biological parents 50. Therefore, even after a supposedly stressful period experienced during a younger age, children were able to adapt a secure attachment with their (second) caregivers, possibly because they were able to incorporate this new and positive experience into their working model, increasing its abilities to cope and deal with stressors. This is consistent with Bowlby's assumption that internal working models are modifiable even after the very first time in life (Bowlby in 50).

The favourable psychological effects of secure attachment are likely mediated by distinct neurobiological processes during development acting on brain circuits involved in emotional and social functioning 60. For example, the *integration* of sensory, emotional and social experiences into the interpretation of one coherent whole, i.e. the integration of right and left hemisphere circuits, are stimulated by attuned parenting behaviour. In support for this, it has been found that maltreatment in early childhood (i.e. experience contrasting secure parenting) disrupts the development of the corpus callosum – a brain structure linking both hemispheres 60. Therefore, early interpersonal experiences (i.e. parenting) contribute to the formation of neurocircuits 52 which enables the child to regulate its emotions effectively 60. Secure attachment is furthermore linked with oxytocin 61. Oxytocin is a neuropeptide that exerts a stress- and anxiety-buffering effect and also interacts with neurotransmitters involved in the reward circuits in the brain 62, possibly further increasing resilience by ‘pre-programming’ the individual to readily perceive rewards and positive emotions.

Through the cascade of positive psychological and neurobiological effects, the development of secure attachment will leave the child more resilient against stressors and negative mental or behavioural outcomes. It helps the individual to effectively cope with aversive events and to regulate behaviours and emotions in appropriate ways 52. It has been shown that if children and caregivers fail to develop a secure attachment, children are more prone to develop mental and behavioural deficits, like externalizing behaviour problems 63, whereas a secure attachment can prevent negative outcomes in children otherwise at risk to develop less optimal outcomes 64. Interestingly, literature relevant to the issue of attachment in the context of parental relationships suggests that the wider social environment may have nurturing qualities such that children growing up in areas with higher levels of social capital or higher levels of social control may have better health outcomes 65.Thus, distinct psychological processes that impact during development on brain circuits involved in emotional and social functioning likely mediate the favourable effects of secure attachment on mental health.

#### Positive emotions

In addition to secure attachment developed early in childhood and the learned ability to trust and love others, evidence suggests that positive emotions are an important source of resilience 66. Studies suggest that positive emotions decrease pain experience and pain catastrophizing 67, 68, while they also seem to contribute positively to health outcomes in general 69. Positive emotions are similarly involved in psychological health recovery. Increases in positive emotion (rather than decreases in negative emotion) during the first week of pharmacological treatment in depressed patients predicted improved depression scores and recovery from depression 6 weeks later 70.

A number of studies indicate that positive emotions protect psychological health by undoing or buffering against the effects of stress. In the laboratory, film clips eliciting positive emotions were associated with faster cardiovascular recovery from a stressful situation than neutral or sad films 71. In daily life, the experience of positive emotions during moments of stress was found to protect psychological health by buffering reactivity of negative emotions to stressful events 72. Moreover, the experience of positive emotions also attenuated the degree to which genetic vulnerability for depression was expressed as a negative mood bias 72. On average, having a twin (especially a monozygotic twin) with a lifetime history of depression was associated with increased negative emotions in response to stressful daily life situations. However, this effect of genetic vulnerability was weakened if there were higher levels of coexperience of positive emotions 72. A similarly protective influence was also found for specific genes. For example, the effect of the BDNF Val66Met polymorphism on social anxiety was much less pronounced when participants experienced high levels of positive emotions 73.

The tendency to experience positive emotions, as measured with questionnaires, has been found to be moderately heritable (*h*^2^ = 0.60) 74. However, when controlling for social and interpersonal stressors, positive emotions were much less heritable, especially in women (0.52 in men, 0.38 in women) 74. This suggests that the person-specific environment may determine to which extent resilience components may actually manifest, and that these differences may also be codetermined by societal influences such as gender socialization. Of note is that overall daily positive emotions (when assessed momentary assessment methods in daily life) are not heritable, suggesting additional person-specific factors at the level of daily life experience impacting on expression of positive emotions (C. Lothmann, N. Jacobs, C. Derom, E. Thiery, J. Van Os, M. Wichers, personal communication). With regard to stability over the course of life, studies suggest that levels of positive emotions are relatively independent of age 75, with a slight decrease in older age 76. Emotional reactivity may be slightly higher during childhood, which may be a critical time window during which caregiver and child learn to fine-tune emotional reactivity through attention and behavioural processes 77. A recent study on gene–environment interactions has found that levels of positive emotions of children with the short (S-) allele of the serotonin transporter gene 5-HTT [compared with the long (L-) allele] were more reactive to differences in parenting style. When raised in a warm and supportive environment, individuals with the S-allele experienced higher levels of positive emotions than individuals with the long (L-) allele. In contrast, they experienced lower levels of positive emotions than individuals with the L-allele when raised in unsupportive environments 78.

Levels of positive emotions vary both between and within people, i.e. emotional experience has enduring (trait-like) and fluctuating (state-like) components. Part of the fluctuation in positive emotions comes from internal (i.e. hormonal) or diurnal influences (i.e. circadian rhythm). Another part arises from interactions in daily life. Meeting up with a friend, playing tennis, or being smiled at in the supermarket are examples of experiences, which may temporarily boost levels of positive emotion. Research has shown that the tendency to use pleasant daily life experiences to boost positive emotions (positive affect reactivity) is associated with increased resilience against depressive symptoms in the future 79, 80. Reward learning mechanisms help an individual to identify pleasant activities and motivate repetition of behaviour associated with increased levels of positive emotions 81. Recent research indicates that, apart from a tendency to generate boosts in positive emotions the tendency to *hold on* to high levels of positive emotions (the duration of the experience) is also associated with improved resilience, expressed as better future recovery of symptoms in depressed patients in response to treatment (Höhn et al. personal communication). Similarly, in individuals recovering from recurrent major depression, a stronger and more lasting inhibitory effect of positive and negative emotions appears to be associated with future recovery 82.

Given the possible major role of positive emotions and the experience of reward in the prevention of and recovery from stress-related disorders, it is important to further disentangle the biological mechanisms of reward experience (see Fig. 2), as this may stimulate new ways of modifying resilience 83. Dopaminergic neuronal transmission in the pathway from the VTA to the NAc has been found to be involved in the responses to both natural rewards like food and sex, as well as unnatural rewards like psychotropic drugs 84. The conscious subjective experience of pleasure and reward likely takes place in the orbitofrontal cortex, which has reciprocal links with the mesolimbic system 85, 86. Animal studies show that the catechol-O-methyl transferase (COMT) enzyme, which breaks down dopamine, is closely involved in dopamine regulation in both subcortical and prefrontal areas, and plays a major role in the dopaminergic signalling exchange between these areas 87, 88. Because dopamine reuptake proteins are sparse in the PFC but not in mesolimbic regions, the COMT enzyme can terminate dopaminergic action in prefrontal synapses, thus exerting a comparably large effect in the PFC 89, which is involved in the conscious experience of pleasure and reward. This implies that lower levels of COMT may be associated with a higher ability to experience reward. Consistent with this proposition, a recent study found that daily life reward experience increased proportionally with the number of Met alleles on the COMT Val158Met polymorphism 90. The Met allele encodes for a less active COMT enzyme, resulting in lower COMT activity, and, as a result, higher levels of prefrontal dopamine 89.

#### Enhancing positive emotions and modulating reward mechanisms

Enhancing the ability to experience positive emotions could play an important role in making people more resilient against depression. A meta-analysis established that positive psychology interventions as diverse as writing gratitude letters, practising optimistic thinking, replaying positive experiences and socializing have beneficial effects on levels of depression 91. Another option may be to give individuals feedback on their own daily life dynamics of emotions. Through the identification of situations associated with positive emotions, people may learn to adapt their behaviour and become more resilient 92, 93.

In addition, meditation- or mindfulness-based approaches may be a promising venue to increase positive emotions. More advanced meditators have been found to possess greater self-awareness and to experience more positive emotions 94, and people have been found to report more positive emotions when in a mindful compared with a non-mindful state 95. In a randomized controlled trial, loving-kindness meditation was associated with increased levels of positive emotion, which in turn predicted reduced depressive symptoms 96. Another randomized controlled trial recently showed that mindfulness-based cognitive therapy (MBCT) was associated with increases not only in positive emotions but also the ability to make use of natural, moment-to-moment rewards in the environment 97. MBCT combines meditation-based techniques 98 with elements of cognitive-behavioural therapy 99, 100. Davidson 101 found that participation in another mindfulness-based therapeutic strategy, i.e. mindfulness-based stress reduction (MBSR), was associated with increased left-sided anterior activation, a pattern consistent with increased positive emotions. Furthermore, an fMRI study showed that loving-kindness meditation regulates neural circuitries (insula, cingulate cortices, amygdala) linked to emotion, theory of mind and empathy 102.

Several studies have shown that mental training is not only associated with behavioural or emotional changes but also leads to observable changes in neural circuits. For example, grey matter volumes in certain areas of the cortex and brain stem differ between novices and experienced meditators (e.g. 103, 104), However, these studies have to be interpreted with caution because of the potentially confounding differences between meditators and novices. Recently, an randomized clinical trial compared participants before and after participation in an 8-week course of mindfulness 105. Even participation in such a short programme was associated with changes in grey matter concentration in brain regions involved in learning and memory processes, emotion regulation, self-referential processing and perspective taking. Overall, results suggest that meditation- or mindfulness-based trainings are promising interventions to increase resilience. Mental training thus leads to a cascade of emotional, behavioural and brain changes associated with resilience. Part of the benefit may stem from facilitation of the reward system, resulting in an increased experience of positive affect.

#### Purpose in life

People may become resilient by experiencing a sense of meaning and life purpose in their lives. In contrast with short-lived momentary pleasures, a sense of life purpose operates at a higher overarching level of experience, which is fed by personal values and individual goals 106. A sense of life purpose may literally keep us alive. Victor Frankl, a concentration camp survivor and psychiatrist, developed a theory predicting the survival chances of his inmates by observing their capacity to find meaning in their current situation. Frankl 107 proposed that any situation can be one in which people find meaning and life purpose, but that psychological problems occur when the search is not successful. Studies showed that the extent to which people experience a sense of life purpose is negatively associated with suicidal ideation 108, 109. Furthermore, experiencing meaning in life was found to buffer against the negative effects of life-threatening physical illness on mental health 110, 111. Loss of meaning and life purpose – through its effects on mental health – indirectly promotes mortality. The concept Sense of Coherence measures the extent to which i) people feel that they understand the things that happen to them, ii) the extent to which they see solutions to problems and iii) the extent to which their daily life is a source of personal satisfaction. In a large population-based cohort Sense of coherence (which conceptually overlaps partly with the concept of ‘purpose of life’) was found associated with slower adaptation to negative life events (Surtees 2006). The latter in turn was associated with increased mortality (Surtees 2006).

Also, religion and spirituality seem to confer resilience 112, 113. Kasen and colleagues 114 report that religious involvement and the reported importance of religion or spirituality in someone's life buffered against the effects of negative life events on mental health. Several potential mechanisms can be hypothesized for this effect. One of them is that religion and spirituality may stimulate a sense of life purpose 112, 115 and that the effects of religion and spirituality on wellbeing may partially be mediated by life purpose experience 115, 116. There is some evidence for an association between spirituality and post-traumatic growth 117, which can possibly be explained by a well-succeeded search for meaning following trauma in highly spiritual individuals.

A state of prayer or sense of union with God or mankind was found to be associated with the activation of several brain areas, under which the left dorsal anterior cingulate cortex, the caudate and the orbitofrontal cortex 118, 119. These are areas that are also implicated in the brain reward system. A speculative thought is that spirituality and experiencing a sense of meaning impact on wellbeing because they serve as facilitators of internal rewards. Every time that people experience sense of meaning in their everyday lives they likely experience strong positive feelings. The continuous availability of internal rewards may make people less dependent on the short-lived external rewards in daily life and may thereby facilitate a healthy level of positive emotions also in the context of adversity.

Thus, religious beliefs may provide a sense of meaning and purpose during difficult life circumstances 112. The conclusion that religion serves as a ‘pervasive and potentially effective method of coping for persons with mental illness’, warrants its integration into psychiatric and psychological practice 112. The current trend of secularization may go hand in hand with decreased population resilience to difficult periods. Therefore, it becomes important to focus on additional sources of sense of meaning and life purpose. Sense of meaning and life purpose is something very person specific and therefore different from behavioural patterns, for which therapists can provide concrete pieces of advice for modification. However, indirectly, prolonged meditation or mindfulness training, in which people are trained to continuously focus their attention to the present moment, may result in increased awareness of meaning and purpose experienced in daily life situations 120.

Besides personal resilience traits, it is also known that the wider social environment has significant impact on resilience outcomes. For example, it has been shown that others have a very important role in the reconstruction of schemas on the self and the world 121, 122 and therefore also on positive emotional experience. Supportive responses from others help individuals to overcome adversity and increase growth following adversity. Parents, family, peers but also characteristics of the school and the neighbourhood may therefore contribute to resilience outcome. Thus, person qualities such as a secure attachment, positive emotions and a purpose of life are imbedded in the context of the impact of the wider social environment on an individual's resilience outcome.

## Discussion

### Linking psychological with biological mechanisms of resilience

Although an extensive number of studies have documented the neurobiological circuitries mediating the stress response and reward experience, it remains a challenge to tease apart the exact biological systems and pathways that mediate and regulate the psychological building blocks (as described above) underlying resilience. This challenge is complicated by the different definitions of the resilience concept used in previous research [see e.g. 6], the different processes (such as sustainability and recovery; of which the psychological and biological underpinnings are, at least partly, distinct from each other) of the concept resilience, and a lack of studies assessing both psychological and biological variables. However, the current state of research does support that the above-mentioned psychological resilience factors in the human studies are related to the stress and reward system of the brain. With respect to attachment, there is some evidence to suggest that experiences of trauma and stress during early life may result in a sensitized stress system, i.e. increased stress responses to smaller stressors such as minor stressors in daily life 123, 124, and that individuals with secure attachment are less stress reactive in adulthood than those without. Similarly, findings from animal studies have suggested that maternal care programmes the offspring's stress responses by epigenetic regulation of gene expression regulating the HPA axis 23, 125, persisting into adulthood. Similar to the observed alterations in methylation level of the promoter region of *NR3C1* in the animal study, a recent study in humans investigated the methylation profile of the *NR3C1* promoter region in postmortem hippocampus samples from suicide victims with a history of childhood abuse, and compared these to the methylation profile in samples from either suicide victims with no childhood abuse or control subjects. Consistent with the animal findings, abused suicide victims had increased CpG methylation of the NR3C1 promoter, concomitant with a decrease in *NR3C1* gene expression 126. Interestingly, the experience of positive emotions during everyday situations seemed to buffer against stress reactivity and against the genetic influence on stress reactivity 72, 127, 128, and it is tempting to speculate that secure attachment may be important in the preprogramming of sensitivity of the reward system, buffering impact of stress systems when activated. The finding that religious practice like praying or remembering a religious experience activated areas of the reward system 118, 119 fits with the hypothesis that positive feelings mediate the process of resilience. One could argue that higher stress sensitivity drives the inability to experience positive feelings, or that stress sensitivity and positive feelings represent two extremes of one and the same continuum. However, a recent study showed that individuals who are stress sensitive in everyday life are not necessarily also low in daily life reward reactivity. In fact, these two phenotypes were not correlated and were not influenced by the same genetic and environmental factors (C. Lothmann, N. Jacobs, C. Derom, E. Thiery, J. Van Os, M. Wichers, personal communication) and thus do not represent the two extremes of a single continuum. This suggests that these traits can co-occur, and may be mediated via different mechanisms. People can be vulnerable in terms of their tendency to be stress reactive, but also protected from this vulnerability trait in the face of strong tendencies to experience positive emotions in daily life (i.e. from pleasant events or sense of meaning) which buffer stress, prevent future psychopathology and increase mental health. Thus, it seems that the experience of positive emotions has a distinct and more central role in resilience defined as the successful adaptation, swift recovery and psychological growth in the face and recovery phase after exposure to severe adversities, while the stress-response systems appears to mainly mediate vulnerability to stressors. Because the stress response and reward systems are closely related both in psychological as well as biological sense (see Fig. 2), it seems very interesting (and challenging) to explore the exact interrelations and crosstalk between psychological and biological factors of the stress response and the reward experience systems. It is therefore interesting that a PET imaging study in adult individuals showed that dopamine release under psychosocial stress in the ventral striatum (where dopaminergic neurons from the VTA project towards) was related to parental care during early life of these individuals 129. More specifically, psychosocial stress caused a significant release of dopamine in the ventral striatum particularly in subjects reporting low parental care, suggesting that resilience to the psychosocial stressor was related to decreased firing of dopaminergic projections from VTA to the ventral striatum (including NAc) 129. Likewise, animals susceptible to the effects of chronic social defeat displayed increased firing rates of dopaminergic VTA neurons, mediated by expression of the *BDNF* gene, whereas unsusceptible or ‘resilient’ animals displayed normal firing rate of dopaminergic VTA neurons, and no behavioural signs of anhedonia in the sucrose preference test (reflecting behaviour related to reward experience) 31, suggesting a crucial role for sustainability of reward experience, mediated by mesolimbic dopaminergic neurotransmission and *BDNF* signalling, in ‘resilience’ to social defeat stress in animals. Other studies have further documented a role for epigenetic changes in the *BDNF* gene and risk for psychiatric disorders, for review see 130. The serotonergic system has furthermore been connected to epigenetic mediation of experience during early life impacting on the stress response system, emotional processing in the brain and affective functioning 131, 132. For example, recent studies on humans and macaque monkeys showed that higher methylation levels of the *5-HTT* gene were associated with stronger effects of stress 133, 134.

It has been proposed that a combination of various adverse environmental exposures throughout development (such as pre-and perinatal stress, low maternal care and childhood trauma) can sensitize the behaviour and central nervous system of an individual, thereby giving rise to a trajectory of risk for psychiatric disorder, starting with subclinical symptoms that become abnormally persistent when synergistically combined with further adversities. Evidence indeed suggests that certain environmental exposures may synergistically lead to subclinical symptoms and subsequent psychiatric disorders by impacting on the HHPA axis 135 and mesolimbic dopaminergic neurotransmission 136 while recent evidence suggests that sensitization to environmental exposures depends on epigenetic mechanisms 20, 137. Together, these findings suggest that experience-dependent regulation of gene expression by the epigenetic machinery impacting on genes involved in the HHPA axis and the mesolimbic dopaminergic reward system underlies the psychological building blocks of resilience by mediating the response and enduring impact of stressors throughout life.

### Current challenges and future perspectives

Despite considerable progress that resilience research has made during the last years, several issues challenge its current state. Progress has been hampered by the use of different definitions and different measures of resilience [for in-depth discussion see e.g. 6], and future studies should better specify the concept of resilience used in the study (e.g. attenuation of health disturbance, or enhanced adaptation and recovery) and how the measured variables of the concept related to these definitions. Resilience studies may thus profit from specifying the definition of resilience, and studying distinct aspects (e.g. recovery) of resilience by incorporating a range of measures that are thought to reflect different levels of resilience such as self-evaluations of functioning, questionnaires and interviews, behavioural and psychological phenotypes, physiological measures such as skin conductance, heart rate and blood pressure and (molecular) biological samples such as salivary cortisol and blood lymphocytes for gene expression and epigenetic analyses. Given difficulties in adequate measurement of psychiatric symptoms and psychological functioning in daily life with regularly used questionnaires and clinical interviews, it may be very productive to extend experience-sampling methodologies (as described above), which are able to capture fluctuations in psychological functioning in daily life in a prospective manner 138.

Because individual trajectories of risk and resilience (Fig. 1) are difficult to capture cross-sectionally at a given moment, and because the available evidence strongly suggest a crucial role for exposures and experiences impacting on development and preadult life on resilience during adulthood, it will be very interesting for future research to prospectively investigate (e.g. birth) cohorts, and to assess the index individuals but also their siblings and parents using genetically sensitive designs. Prospective twin studies will be very informative in teasing apart the contributions of genetic factors, environmental factors and gene–environmental interactions. Given recent preliminary findings suggesting a crucial role of the epigenetic machinery in regulating adaptive responses to stress (described above), further research may establish the role of epigenetics in resilience, for example, by studying monozygotic twins discordant for resilience-related phenotypes.

To optimally align the translational aspects of human and animal studies on resilience, it will further be important to use (or design) behavioural and biological tests that can be conducted in both research settings. For example, measures of social approach and avoidance behaviour, generalization of anxiety, measures of mother–child relationships, sensitivity of the autonomous nervous system to a standardized stressor, or the cortisol/corticosterone response to a standardized stressor is possible in various animal species and may be very useful from a translational neuroscience perspective.

The field of experimental animal research may be particularly fruitful in elucidating the molecular and cellular mechanisms of resilience when extending its focus from mere investigations on the impact of a stressor by comparing exposed vs. non-exposed groups, to also studying differential susceptibility to a given stressor 139 as well as studying the rate of recovery of animals showing stress-related behavioural disturbances.

### Recommendations for interventions aimed at increasing resilience in humans

The current literature review suggests that positive emotions are crucial to counteract stress experience. Feelings of positive emotions are strongly related to sense of meaning and life purpose. Interventions that successfully increase the experience of positive emotions have become available in western society 96, 97. Meditation techniques such as Loving-kindness and mindfulness training may both increase feelings of purpose of life together with positive emotional experience. These effects have been established both at the psychological and the biological level as mental training through meditation has been shown to change brain function 140. Also, ancient religious practices, such as praying, counting one's blessings and finding oneness with God contribute to sense of meaning and positive emotional experience 116, 118, 119. Engaging in religious practices may thus actually have a positive influence on one's level of resilience. This fact should be further acknowledged and understood in current practices of mental health care to optimally support patients in their search for meaning.

To conclude, the current literature on resilience does show some converging evidence on links between psychological and biological aspects at the individual level although the field is expected to greatly benefit in the near future from multidisciplinary and translational-oriented research efforts.
